# Phylogenomic diversity and chromatic acclimation in phycoerythrin-containing Microcystis aeruginosa

**DOI:** 10.1099/mgen.0.001852

**Published:** 2026-09-29

**Authors:** Yuuhiko Tanabe, Haruyo Yamaguchi, Tomoharu Sano

**Affiliations:** 1 Biodiversity Division, National Institute for Environmental Studies, Tsukuba, Japan; 2 Center for Environmental Measurement and Analysis, National Institute for Environmental Studies, Tsukuba, Japan

**Keywords:** chromatic acclimation, cyanobacteria, ecotype, microcystis, phycoerythrin, phylogenomics

## Abstract

*Microcystis aeruginosa* is a bloom-forming cyanobacterium widely distributed in eutrophic freshwater systems. Most strains are blue–green due to the presence of the photosynthetic pigments phycocyanin and chlorophyll *a*. Less commonly, brownish strains have been reported, reflecting the presence of the red pigment phycoerythrin (PE). However, the genomic basis, phylogeny and evolutionary origin of PE pigmentation in *M. aeruginosa* remain poorly understood. Here, we characterized PE-containing *M. aeruginosa* strains, including six newly sequenced genomes. All genomes harboured putative PE synthesis and regulatory genes (the *cpe* cluster). Phylogenomic analysis further revealed that these PE-containing strains are distributed across three distinct phylogenetic groups. In contrast, the genomic organization of the *cpe* cluster was largely conserved. Spectroscopic analyses further indicated that strains belonging to different phylogenetic groups exhibit the same type of chromatic acclimation. Moreover, the phylogenetic relationships inferred for each PE gene were congruent and consistent with the whole-genome phylogeny. Our findings support a single origin of PE genes, with the observed distribution best explained by two independent horizontal transfer events rather than repeated gene loss during intraspecific diversification in *M. aeruginosa*.

**Impact Statement**

Phycoerythrin (PE) is an accessory photosynthetic pigment that complements chlorophyll and is widely distributed among cyanobacteria, including the bloom-forming *Microcystis aeruginosa*. However, PE-containing *Microcystis* strains are relatively rare and remain poorly characterized. By generating and analysing whole-genome sequences of PE-containing strains, this study provides the first genomic insights into the basis of PE pigmentation and the mechanisms underlying light acclimation in *Microcystis*. Our analyses further reveal the distribution of PE-containing strains across the diverse phylogeny of *Microcystis*. In addition, we propose an evolutionary scenario for PE biosynthesis genes in this genus, suggesting a key role for horizontal gene transfer. These findings advance our understanding of the evolution and functional integration of PE in cyanobacteria. They also provide a foundation for future studies on the ecological significance of PE-containing *Microcystis*, particularly in nutrient-rich aquatic environments where this genus frequently proliferates.

## Data Summary

The authors confirm all supporting data and protocols have been provided within the article or through supplementary data files. Accession numbers for the six genome datasets generated in this study are listed in Table S1 (available in the online Supplementary Material). These data are available in the DNA Data Bank of Japan (DDBJ)/European Molecular Biology Laboratory (EMBL)/GenBank databases under the accession numbers GCF_008579225, GCF_008579565, GCF_008579685, GCF_008579325, GCF_008579445 and BAAFSX010000001.

## Introduction

*Microcystis aeruginosa* sensu [1] (hereafter denoted *Microcystis*) is one of the most common bloom-forming cyanobacteria found in fresh and brackish waters worldwide [2]. *Microcystis* bloom occurrences in lakes and reservoirs cause several environmental problems, including foul odour and bottom-layer hypoxia. However, the production of hepatotoxic cyanotoxins, microcystins, represents a major concern [2]. Cases of intoxication of humans [3], livestock [4] and wild animals [5] by microcystin contamination have been sporadically reported across diverse regions.

In most cyanobacteria, including *Microcystis*, the water-soluble macromolecular complex phycobilisome is associated with thylakoid membranes and functions as a light-harvesting apparatus that complements photosystem II. The primary function of phycobilisomes is to enable cells to absorb light in the 550–600 nm wavelength range, which cannot be efficiently absorbed by chlorophyll molecules. This capacity facilitates photosynthetic growth under conditions where longer-wavelength light is limited or unavailable, such as in deep underwater environments [6]. Phycobilisomes consist of a core (allophycocyanin), rod phycobiliproteins such as phycocyanin (PC) and phycoerythrin (PE) and linker polypeptides that unite the cores and rods [7]. The type and the number of rod phycobiliproteins incorporated into the phycobilisome mainly determine the colour of the cyanobacterial cells [8]. Several cyanobacterial species and strains are known to alter their colouration in response to the spectral quality of available light, for example by replacing rod phycobiliproteins with alternative ones. This process is called chromatic acclimation (CA) [9, 10]. CA can be categorized based on the different modes of phycobiliprotein accumulation. Type I (CA1) strains show no change in the content of both PE and PC; type II (CA2) strains show an increase in PE but not in PC content under green light; type III (CA3) strains increase both PC and PE content under red and green light, respectively [10]. Other complex types of CA include a change in phycobilin content in the chromophore in response to blue/green light (CA4) and dynamic remodelling of phycobilisomes under far-red light perception [9].

Like other cyanobacteria, most *Microcystis* strains are characterized by a blue–green colour owing to the presence of photosynthetic pigments: chlorophyll *a* and PC [11]. *Microcystis* strains with greyish to dark brown pigmentation have also been isolated, albeit rarely 11–13]. Previous studies have suggested that the brownish colouration of *Microcystis* is attributable to the presence of PE, in addition to PC and chlorophyll *a *[11, 13]. Phylogenetic analyses based on 16S rDNA and Internal Transcribed Spacer (ITS) sequences have further indicated that PE-containing strains are genetically highly similar to PE-negative strains of *Microcystis* [14], suggesting that this variation reflects intraspecific diversity rather than species-level differentiation [15]. However, PE pigmentation in *Microcystis* has not been extensively characterized within a phylogenetic framework. This may partly reflect the limited availability of PE-containing strains suitable for in-depth phylogenetic analysis. Genes involved in PE synthesis and regulation in *Microcystis* have not been characterized in detail. It is also unknown whether PE-containing *Microcystis* strains regulate their PE content in response to different light wavelengths and undergo CA. In addition, due to limited information on PE genes in *Microcystis*, their evolutionary history within the species remains unclear.

To address these questions, we characterized brownish *Microcystis* strains using whole-genome sequencing, phylogenetic analyses and phycobiliprotein profiling. Our results showed that PE-containing strains do not form a monophyletic group. Nevertheless, the genes involved in PE biosynthesis and regulation, as well as their genomic organization, were highly conserved among PE-containing *Microcystis* strains. Based on these findings, we discuss potential scenarios underlying the evolution of PE pigmentation in *Microcystis*.

## Methods

### Strains

*M. aeruginosa* Ks07TS04 and HCR1709 were isolated from bloom samples collected from Lake Kasumigaura, Japan (N 36.079558, E 140.209157), on 3 August 2007, and Lake Hachiro, Japan (N 40.08668, E 139.99966), on 22 September 2017, respectively using a micropipetting isolation method as described previously [16]. Additionally, the strains Ki05YA01, Tn05Ak01 [16], Ks07TS139, Hs07SP05 [17] and NIES-1211 [18] were used for subsequent analyses. All seven strains appeared brownish in culture under white light, suggesting the presence of PE. Strains Ks07TS04, Ki05YA01, Tn05Ak01, Ks07TS139 and Hs07SP05 were deposited at the Microbial Culture Collection of the National Institute for Environmental Studies (MCC-NIES, Tsukuba, Japan) under strain no. NIES-4264, NIES-2519, NIES-2520, NIES-2521 and NIES-2522, respectively. Additionally, a PE-negative strain NIES-4234 (=Sj) [18] was used as a reference for spectral analysis of phycobiliproteins.

### Whole genome shotgun analyses

*M. aeruginosa* NIES-2519, NIES-2522, NIES-4264 and HCR1709 were not axenic. Bacterial contaminants were removed from these samples prior to DNA extraction according to a previously published protocol using repeated filtration [18]. Genomic DNA of each strain was extracted using NucleoBond^®^ AXG Columns with Buffer set III (Macherey-Nagel, Düren, Germany). DNA was fragmented to ~450 bps using Covaris M220 (Covaris, Woburn, MA). A 450 bp fragmented library was constructed using the NEBNext Ultra DNA Library Prep Kit for Illumina (New England Biolabs, Ipswich, MA). DNA was sequenced using the MiSeq platform (Illumina, San Diego, CA) with the 500-cycle MiSeq Reagent Kit v2 and Nano Kit v2. *De novo* assembly of the contigs was performed using Spades ver. 3.10.1 [19] and automated annotations were performed using Prokka ver. 1.12 [20] or dFAST [21]. Contigs of possible contaminants were excluded based on lower coverage (identified by Spades) and a blast analysis. The HCR1709 genome assembly exhibited substantial contamination from another heterotrophic bacterium; therefore, genome binning was performed using MaxBin 2.0 [22]. The whole-genome shotgun datasets of NIES-2519, NIES-2520, NIES-2521, NIES-2522, NIES-4264 and HCR1709 have been submitted to the DDBJ/EMBL/GenBank databases (Table S1).

### Phylogenomic analyses

RefSeq genome data of *Microcystis* and broad representative cyanobacteria were retrieved from GenBank with reference to Strunecký *et al.* [23]. Although more than 500 *Microcystis* genomes are currently available in GenBank (as of 22 April 2026), we restricted our analysis to RefSeq entries to simplify the graphical representation of the phylogenomic tree of *Microcystis*. This approach does not affect our conclusions, given the limited number of PE-positive strains. Maximum-likelihood (ML) phylogenomic trees were reconstructed using RAxML version 8.2.12 [24] through PhyloPhlAn version 3.1.68 [25], based on an amino acid alignment of 400 universal marker genes defined in PhyloPhlAn [26] and applying the PROTGAMMA substitution model. The ‘--diversity low’ and ‘--diversity medium’ options were used for the reconstruction of the *Microcystis* and global cyanobacterial phylogenies, respectively. ML bootstrap analyses were performed using 100 random replicates. Genome sequences used for phylogenomic reconstruction are provided in Tables S1 and S2.

### Phylogenetic analyses

Phylogenetic analyses of PE gene sequences were conducted using the neighbour-joining method with 1,000 bootstrap resamplings, applying the maximum composite likelihood substitution model with uniform nucleotide substitution rates among sites and lineages to calculate distances between strains, as implemented in mega version X [27]. Aa alignments of CpeA and CpeB were generated using muscle [28] in mega, and gap positions were removed from the alignments. An ML phylogenetic tree was subsequently reconstructed using RAxML based on the concatenated aa sequences of CpeA and CpeB (336 aas), employing the LG+I+G4+F model selected by ModelTest-NG [29]. Sequences used in the phylogenetic reconstructions were indicated in Tables S3 and S4.

### Spectral analyses

An aliquot of 2 ml of mid-exponential-phase *Microcystis* cultures grown in liquid MA medium [30] under white light was inoculated into 2 ml of fresh MA medium and cultivated in 20 ml screw-cap test tubes at 23–25 °C under white, monochromatic green (λ_max_=525 nm) and red (λ_max_=660 nm) LED lights (24 h, 10 µmol photons m^−2^ s^−1^) using ISL-150×150 (CCS Inc., Kyoto, Japan). After 1 week, *in vivo* absorption spectra were measured using a spectrophotometer UV-1800 (Shimadzu, Kyoto, Japan). For *in vitro* absorption spectra, 1 ml culture was sonicated for 5 min and centrifuged at 20,000 ***g*** for 5 min. The pellet was suspended in 1 ml of 1× PBS buffer (pH 7.2) in a microtube. Zirconium beads (0.1 mm diameter) were added to the cell suspension, and the cells were disrupted using a Mini Bead-Beater (Biospec Products, Bartlesville, OK) at 4,200 r.p.m. for 60 s. The lysate was then centrifuged at 20,000 ***g*** for 10 min. Supernatants were used for measurement of absorption spectra.

## Results and discussion

### Phylogeny of PE-containing strains of *Microcystis*

We obtained draft genome sequences of *Microcystis* strains, presumed to contain PE based on their brownish pigmentation under white light (Fig. S1). As expected, all brownish strains used in this study (NIES-1211, NIES-4264, NIES-2519, NIES-2520, NIES-2521, NIES-2522 and HCR1709) harboured a putative gene set involved in PE synthesis and regulation (hereafter designated the ‘*cpe* cluster’) (Tables S4). Based on this finding, these strains are hereafter referred to as PE-containing strains. blast searches indicated that the *cpe* cluster is extremely rare in the *Microcystis* genome. Among more than >500 published genomes, only two additional *Microcystis* strains (MC19 and T1-4) harbour the *cpe* cluster (Tables S1 and S4). Phylogenomic analysis of *Microcystis* indicated that PE-containing strains are not monophyletic but are distributed across three distinct phylogenetic groups (A1, G and K) (Fig. 1). The non-monophyly of PE-containing strains is consistent with an earlier ITS-based study, which also placed these strains, albeit with low resolution, in different phylogenetic groups [15]. Given the substantial genetic divergence among PE-containing strains within group A1 and within group K, these strains likely represent stable PE-containing ecotypes. In contrast, the PE-containing NIES-2521 represents an orphan lineage (a singleton) in group G. This suggests either that the acquisition of PE by NIES-2521 was a recent event, or that its apparent uniqueness may be an artefact of limited strain sampling.

**Fig. 1.**
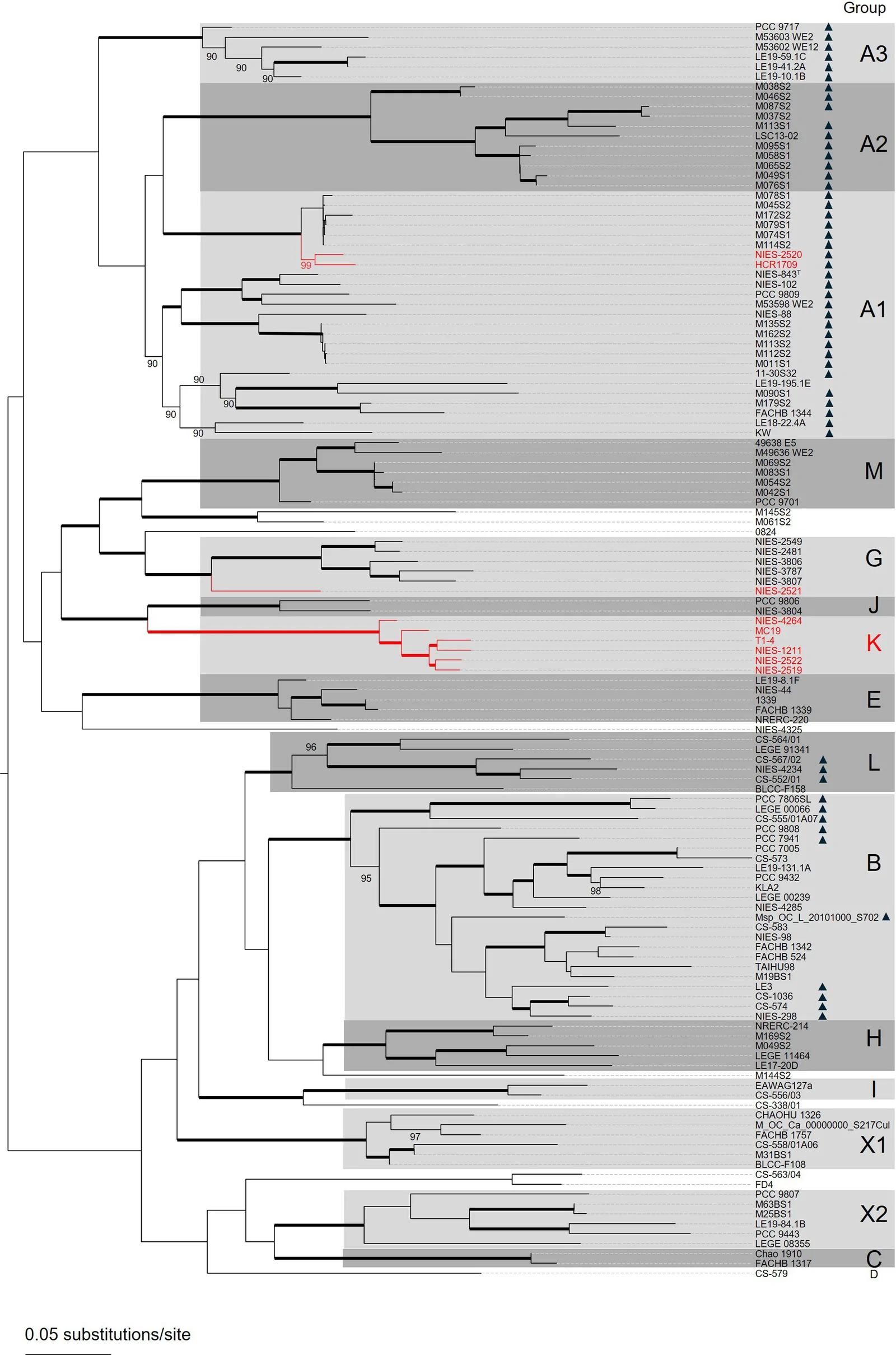
Phylogenomic tree of 195 *Microcystis* genomes. Intraspecific phylogenetic groups were defined according to a previous report [18], with newly designated groups K, L and M. Based on the substantial divergence, groups A and X were subdivided into three and two groups, respectively. Numbers at the major branches indicate RAxML bootstrap values. Branches with statistical support (RAxML BP=100) are shown in bold. PE-containing strains and lineages are shown in red. Triangles denote strains positive for microcystin genes [47].

### PE genes in *Microcystis*

In all currently available strains, the *cpe* cluster was detected on two separate contigs (Fig. 2). Determining whether the cluster is physically separated in the genome requires complete genome sequences. Based on homology to previously characterized PE genes from other cyanobacteria, each gene in the *cpe* cluster can be annotated. These include genes for the alpha and beta chain of PE (*cpeA* and *cpeB*), PE-associated linker proteins (*cpeC* and *cpeD*), phycoerythrobilin (PEB) synthesis (*pebA* and *pebB*), PE lyase (*cpeS*, *cpeT* and *cpeZ*) and an activator for *cpe* expression (*cpeR*) [31]. Notably, a green/red light sensor histidine kinase gene (*ccaS*) and its cognate response regulator gene (*ccaR*) are shared in the cluster. Under green light, CcaS phosphorylates CcaR, enabling its binding to the PE gene promoter and thereby activating PE gene expression. In contrast, under red light, CcaR is dephosphorylated, resulting in its dissociation from the promoter and consequent repression of PE gene expression [32]. The presence of *ccaS* and *ccaR*, despite their distinct genomic contexts, suggests that all PE-containing *Microcystis* strains share a common molecular basis for CA2.

**Fig. 2.**
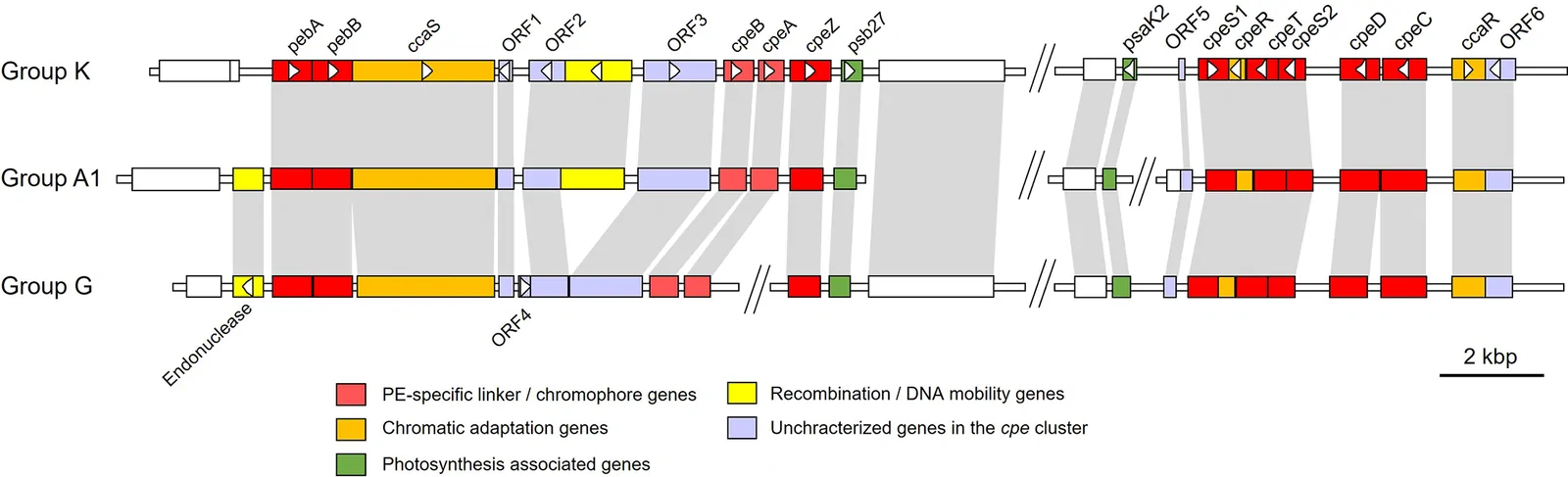
Organization of genes functioning in PE synthesis and regulation in the genome of PE-containing *Microcystis* strains. Grey shading indicates homologous genes. Double slashes indicate non-contiguous regions (from separate contigs); intervening sequences are omitted for clarity.

To confirm CA2 in PE-containing *Microcystis*, spectral analyses were performed. Under red light, cultures of three PE-containing strains representing phylogenetic groups A1, G and K appeared green (Fig. 3a). Consistent with visual inspection and genome analyses, the *in vivo* absorption spectra of the brownish strains exhibited a dominant peak at ≈573 nm, indicative of PE, whereas this peak was absent in the blue–green strain NIES-4234 (Fig. 3b). *In vitro* spectra showed pronounced changes in the PE absorption peak under varying green/red light conditions, whereas the PC peak changed only slightly (Fig. 3c–e), suggesting that all three strains represent type II chromatic acclimators (CA2 [10]).

**Fig. 3.**
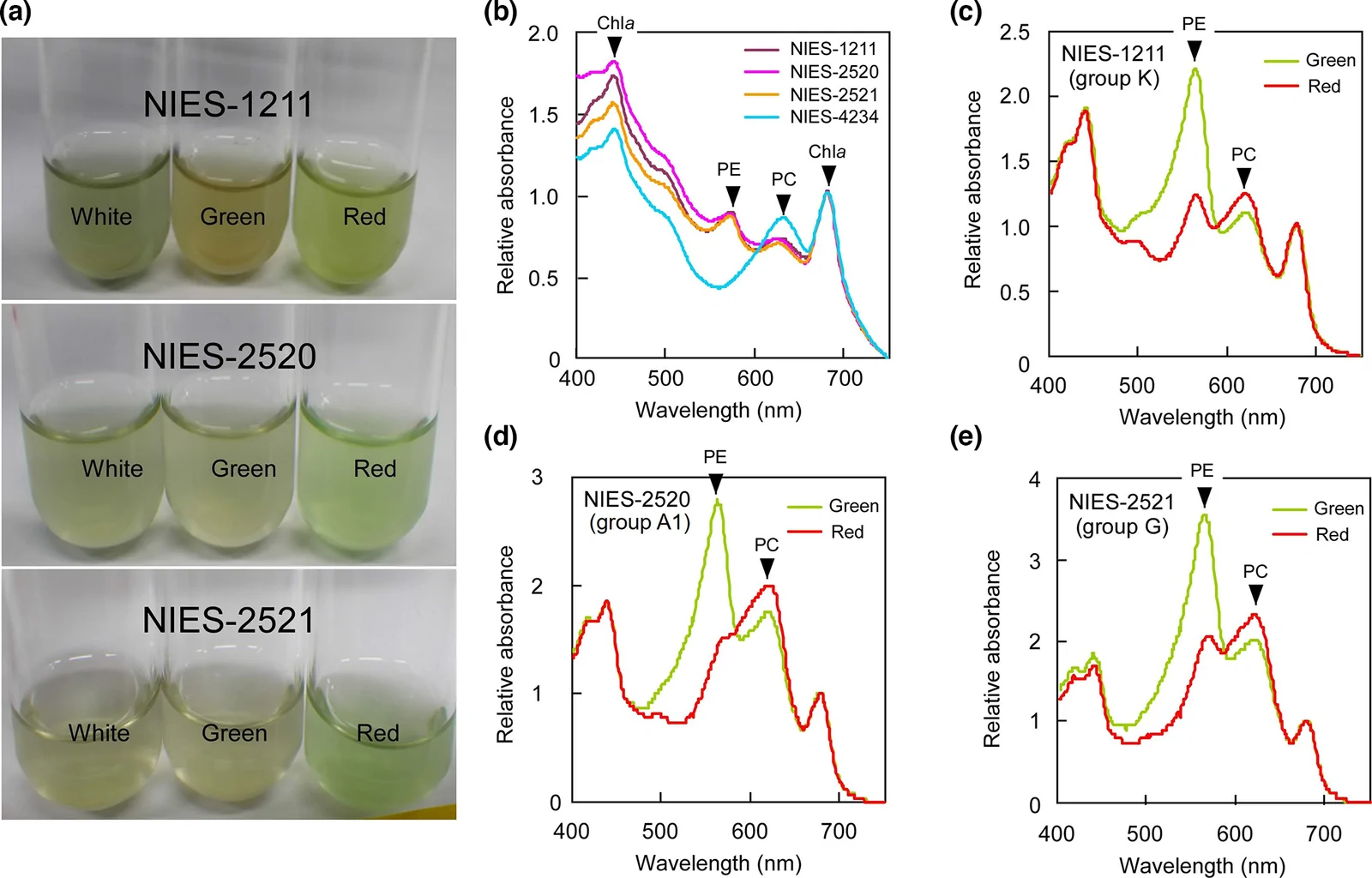
(a) PE-containing *Microcystis* strains (NIES-1211, NIES-2520 and NIES-2521) representing different phylogenomic groups (K, A1 and G, see Fig. 1) grown under white, green and red light. Note that all three strains turned green in colour under red light, indicative of CA. (b–e) Absorption spectra. (**b)** *In vivo* spectra of *Microcystis* strains grown under white light (full spectrum); a PE-deficient strain NIES-4234 (Fig. S1) is used as a control. *In vitro* spectra of (**c)** NIES-1211; (**d)** NIES-2520; (**e)** NIES-2521. The *in vitro* spectra also exhibit peaks attributable to chlorophyll *a* contamination. All spectra were normalized using the chlorophyll *a* absorption peak at 680 nm.

In addition to *ccaS*/*ccaR*, genes for another putative sensor His-kinase (ORF3) and response regulator (ORF2) were found in the *cpe* cluster. Although the functions of these proteins remain unknown, ORF3 is unlikely to be a CcaS homologue because it lacks the cGMP-specific phosphodiesterases, Adenylyl cyclases, and FhlA (GAF) domain essential for cyanobacteriochrome photoreceptor function [33]. The gene organization within the *cpe* cluster is highly conserved across all PE-containing strains (Fig. 2). Minor differences include insertions or deletions of genes encoding putative transposases, endonucleases (possibly involved in genome rearrangement) and small ORFs. blast searches indicated that all genes in the *cpe* cluster are confined to this cluster. These observations suggest a shared common ancestry of the *cpe* clusters in *Microcystis*.

### Phylogenetic distribution and molecular evolution of PE genes

Genes underlying adaptive traits in *Microcystis* have frequently undergone horizontal gene transfer (HGT) (e.g. salt resistance [18, 34]). This raises the possibility that HGT contributes to the patchy distribution of PE genes in *Microcystis*. To test this hypothesis, phylogenetic analyses of PE genes were performed. A phylogenetic analysis across a broad range of cyanobacteria (Fig. 4) revealed significant discordance between genome and CpeAB phylogenies.

**Fig. 4.**
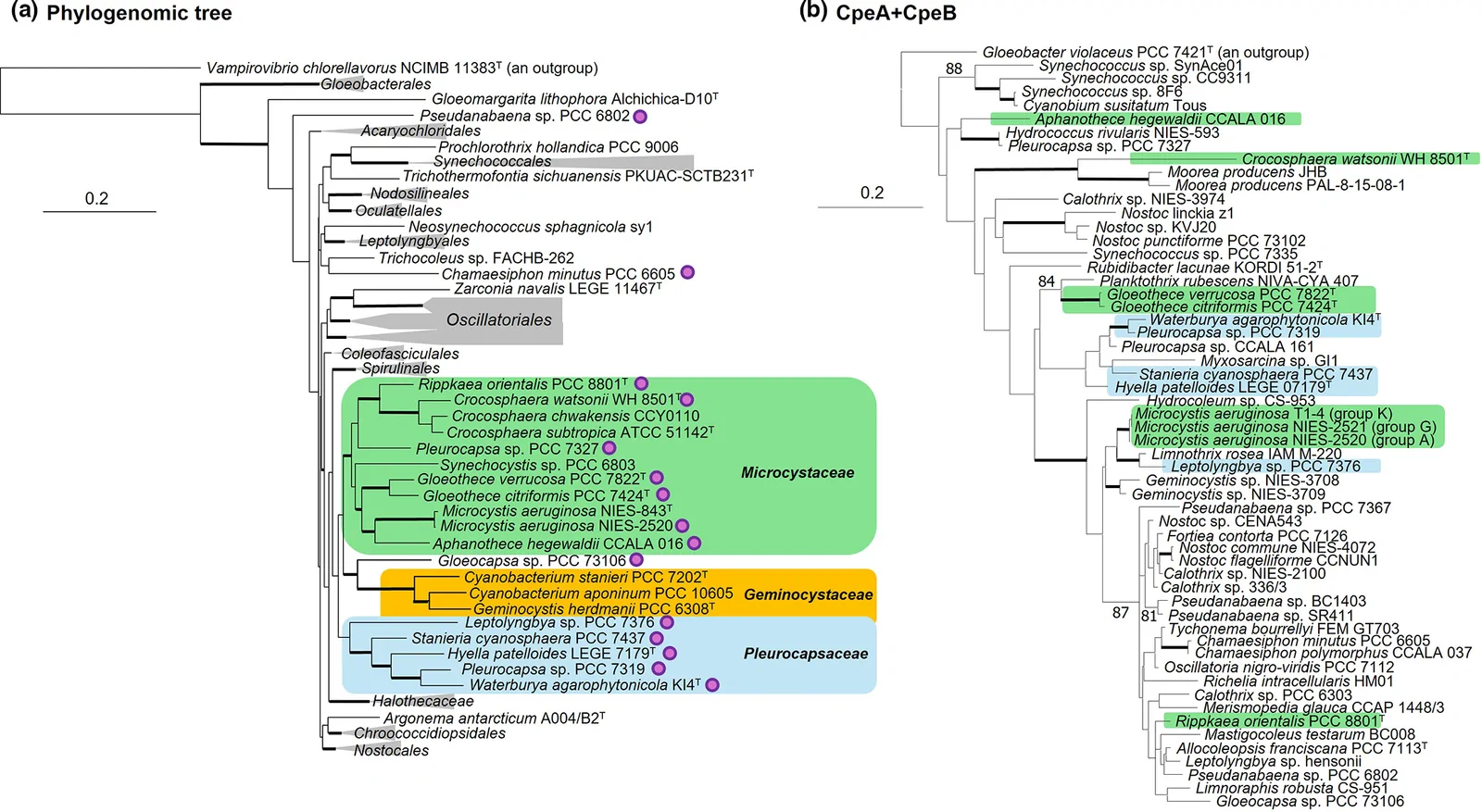
Molecular phylogenies of Cyanobacteria. Branches with statistical support (RAxML BP >95) are shown in bold. (**a)** Phylogenomic tree of Cyanobacteria. NIES-2520 was included as a representative of PE-containing *Microcystis* strains. Statistical support for key branches is indicated at the corresponding nodes. PE-containing strains are indicated by purple circles. (**b)** Phylogenetic tree of concatenated aa sequences of CpeA and CpeB (336 aas in total). Scale bar, substitutions per site.

For example, strains within the *Microcystaceae* form a monophyletic clade in the phylogenomic tree but are dispersed across the CpeAB phylogeny. This result suggests that HGT of PE genes occurred frequently during the diversification of Cyanobacteria. The CpeAB phylogeny further indicated that PE genes in *Microcystis* are monophyletic. These results suggest that PE genes in *Microcystis* share a common origin, likely acquired via HGT from another cyanobacterium. However, identifying the donor was challenging due to the substantial genetic divergence between *Microcystis* and its closest relatives (i.e. *Leptolyngbya* sp. PCC 7376 and *Limnothrix rosea* IAM M-220). Phylogenies of individual PE genes in *Microcystis*, including regulatory genes (e.g. *ccaS*), were largely congruent with the genome phylogeny, indicating overall consistency (Figs 5 and S2). This suggests that the *cpe* cluster was horizontally transferred as an intact unit, rather than through partial transfer of individual genes or subclusters.

**Fig. 5.**
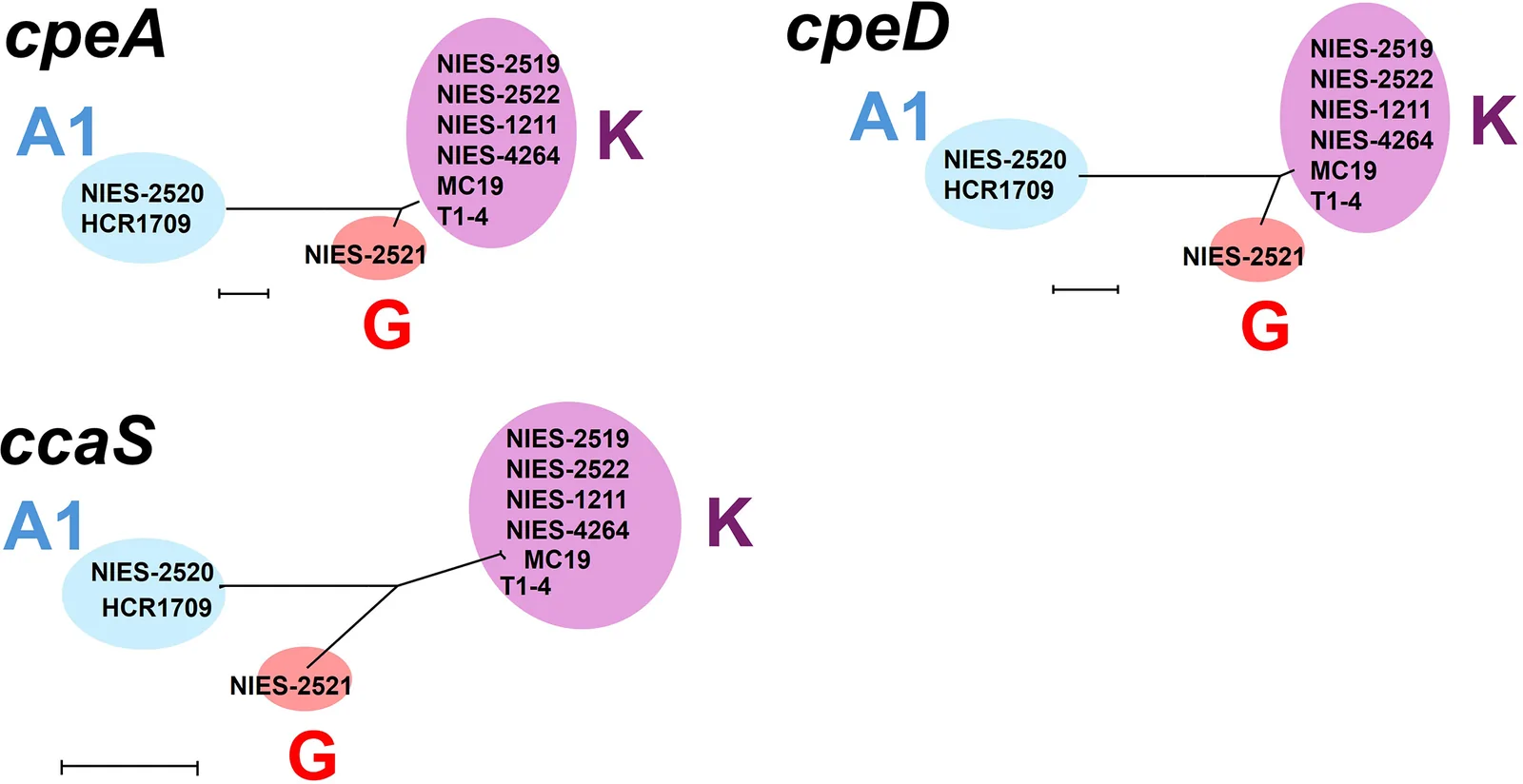
Unrooted trees based on nucleotide sequence of *cpeA* (495 bps), *cpeD* (768 bps) and *ccaS* (2,694 bps). Scale bar, 0.01 substitution per site.

Two hypotheses may explain the observed pattern of PE gene divergence in *Microcystis*. First, PE genes may have been introduced into *Microcystis* via early HGT and subsequently lost divergence into the three intraspecific groups A1, G and K (‘PE-early’). However, this scenario appears less likely, given that most extant *Microcystis* genotypes lack PE. Alternatively, recurrent HGT may have driven the spread of PE genes among *Microcystis* prior to the divergence of groups A1 and K (‘PE-late’). We favour the latter scenario, as the observed distribution of the *cpe* gene cluster is more parsimoniously explained by two transfer events than by at least eight independent losses. This interpretation is further supported by the conservation of gene organization across strains and the presence of mobile genetic element–related genes both within and in the flanking regions of the *cpe* gene cluster (Fig. 2) [[35]]. Although compositional analyses such as GC content are uninformative in this case due to the similarity among strains, the combined evidence supports horizontal gene transfer as the most likely evolutionary scenario. Consistent with this interpretation, intraspecific HGT of the PE gene cluster has also been reported in the bloom-forming cyanobacterium *Planktothrix* [36]. A combination of these two hypotheses may explain the current distribution of PE genotypes. Nevertheless, the *cpe* cluster in *Microcystis* clearly originated from a single ancestor and was introduced early in the evolution of this species. *Microcystis* genome is known to be highly plastic [34, 37, 38]. Gene content variation underlying genome plasticity in *Microcystis* includes genes involved in secondary metabolism [39], compatible solute synthesis [18] and inorganic carbon uptake [40]. Gain and loss of PE genes during *Microcystis* diversification provide an additional example of genome plasticity associated with photosynthesis.

### Ecological and evolutionary implications

The current data suggest that PE-containing strains are divided into three phylogenetic groups. Group K are the most diverse PE-containing lineage and likely represent a stable, persistent ecotype [41]. This suggests that the presence of PE in the phycobilisome confers an ecological advantage over PE-negative *Microcystis* strains within their ecological niche. A classic consideration points to the advantage of PE over non-PE pigmentation; deep underwater environments, where longer wavelengths of light are limited, require phototrophs to use green light efficiently [6]. However, *Microcystis* does not appear to preferentially inhabit deeper, green light–enriched waters. Instead, it exhibits vertical migration, moving from deeper layers for nutrient acquisition to the surface for light harvesting. This movement is regulated by the balance between gas vesicle protein (Gvp) expression and intracellular accumulation of photosynthetic carbohydrates [42]. Indeed, the six PE-containing strains (NIES-2519, NIES-2520, NIES-2521, NIES-2522, NIES-4264 and HCR1709) were isolated from surface waters. blast analyses further showed that all PE-containing *Microcystis* strains harbour Gvp genes encoding Gvps [43]. These observations suggest that the PE-containing strains do not preferentially inhabit deeper water layers. PE pigmentation *in Microcystis* can be explained by spectral partitioning of light, which facilitates the coexistence of phytoplankton with different wavelength preferences and drives adaptive pigment divergence [44]. This theory is supported by studies of natural waters, which suggest that intermediate turbidity favours the coexistence of different pigment types even in shallow environments [45]. This is consistent with the frequent coexistence of both PE-positive and PE-negative genotypes within the same *Microcystis* bloom. For example, NIES-2521 and other PE-negative group G strains have been co-isolated from the same bloom in eutrophic shallow water in Lake Kasumigaura, where the maximum depth is 7 m [16]. A field survey of Lake Kasumigaura further demonstrated the coexistence of PE-positive and PE-negative genotypes, although PE-positive genotypes were infrequently observed (Fig. S3). Comprehensive field surveys of the spatial distribution of PE-containing *Microcystis* strains, coupled with laboratory co-culture experiments, would provide further insights into the coexistence of PE-positive and PE-negative genotypes. In an evolutionary context, the observed coexistence of PE-positive and PE-negative *Microcystis* strains likely reflects adaptive radiation mediated by interspecific HGT of PE genes. Similar patterns have been reported in marine cyanobacteria such as Synechococcus [8, 46].

## Supplementary material

10.1099/mgen.0.001852 Supplementary Material 1.

10.1099/mgen.0.001852 Supplementary Material 2.

## Abbreviations

CA: chromatic acclimation
Gvp: gas vesicle protein
HGT: Horizontal gene transfer
MCC-NIES: Microbial Culture Collection of the National Institute for Environmental Studies
ML: maximum-likelihood
PC: phycocyanin
PE: phycoerythrin
PEB: phycoerythrobilin
