# Supplementary material for: Phylogenomic diversity and chromatic acclimation in phycoerythrin-containing Microcystis aeruginosa
Source: Microb Genom. 2026 Sep 29;12(9):001852. doi: 10.1099/mgen.0.001852 (PMC13623022; doi:10.1099/mgen.0.001852)
Supplement: Supplementary file 1 [file mgen-12-01852-s001.pdf]

Phylogenomic diversity and chromatic acclimation in phycoerythrin-containing *Microcystis aeruginosa*

Yuuhiko Tanabe*[1], Haruyo Yamaguchi[1], Tomoharu Sano[2]
[1]Biodiversity Division, National Institute for Environmental Studies, Japan
[2]Center for Environmental Measurement and Analysis, National Institute for Environmental Studies, Japan

*For correspondence: Yuuhiko Tanabe

Biodiversity Division, National Institute for Environmental Studies, Japan
16-2 Onogawa, Tsukuba, Ibaraki 305-8506, Japan
TEL: +81-29-850-2556 FAX: +81-29-850-2587
E-mail: tanabe.yuuhiko@nies.go.jp; ytanabehiko@gmail.com

Supplementary Figures

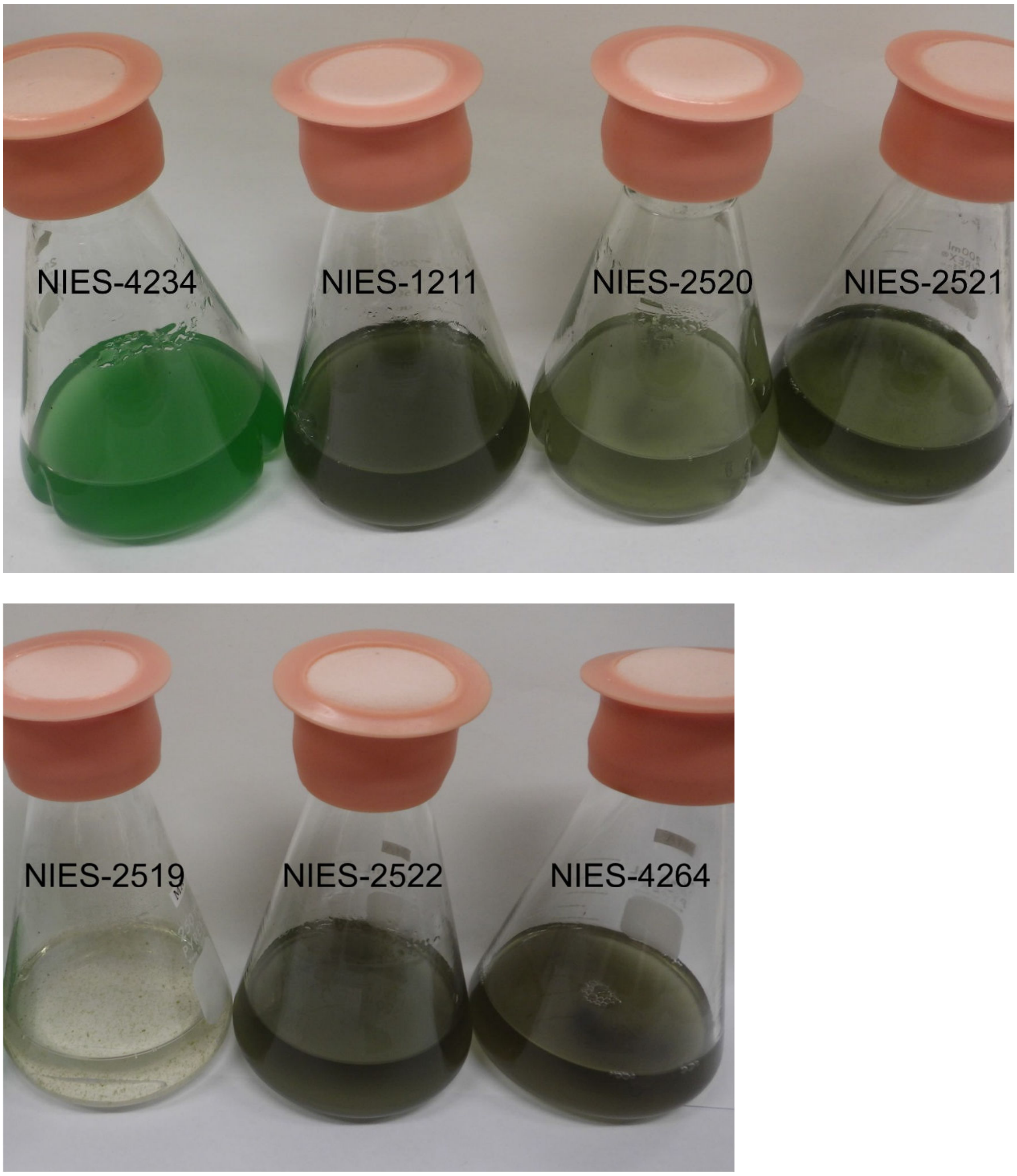

Fig. S1 Cultures of PE-containing *Microcystis* strains and one PE-deficient strain (NIES-4234) used in this study.

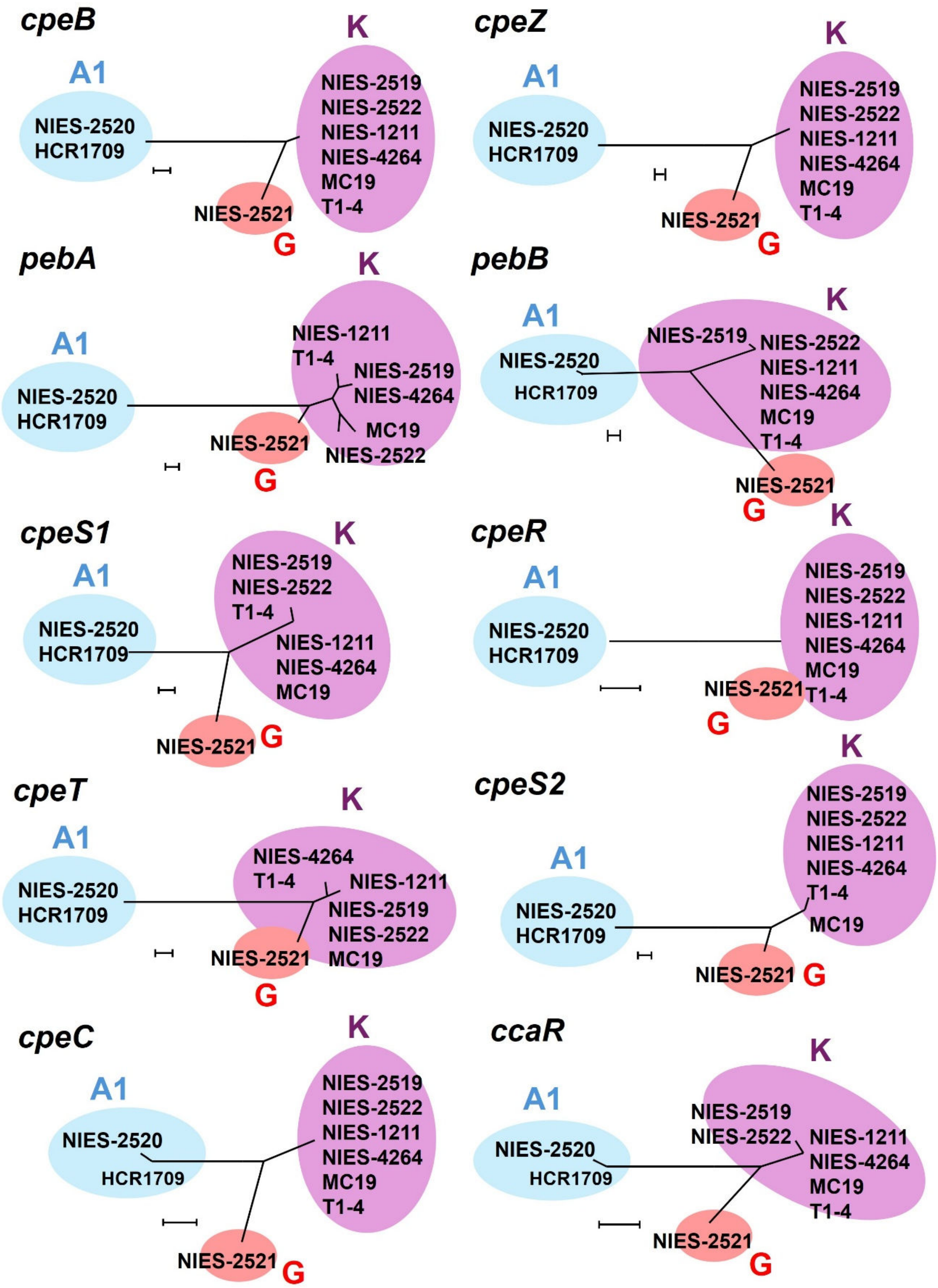

Fig. S2 *cpe* phylogenies. Scale bar: 0.002 substitutions per site.

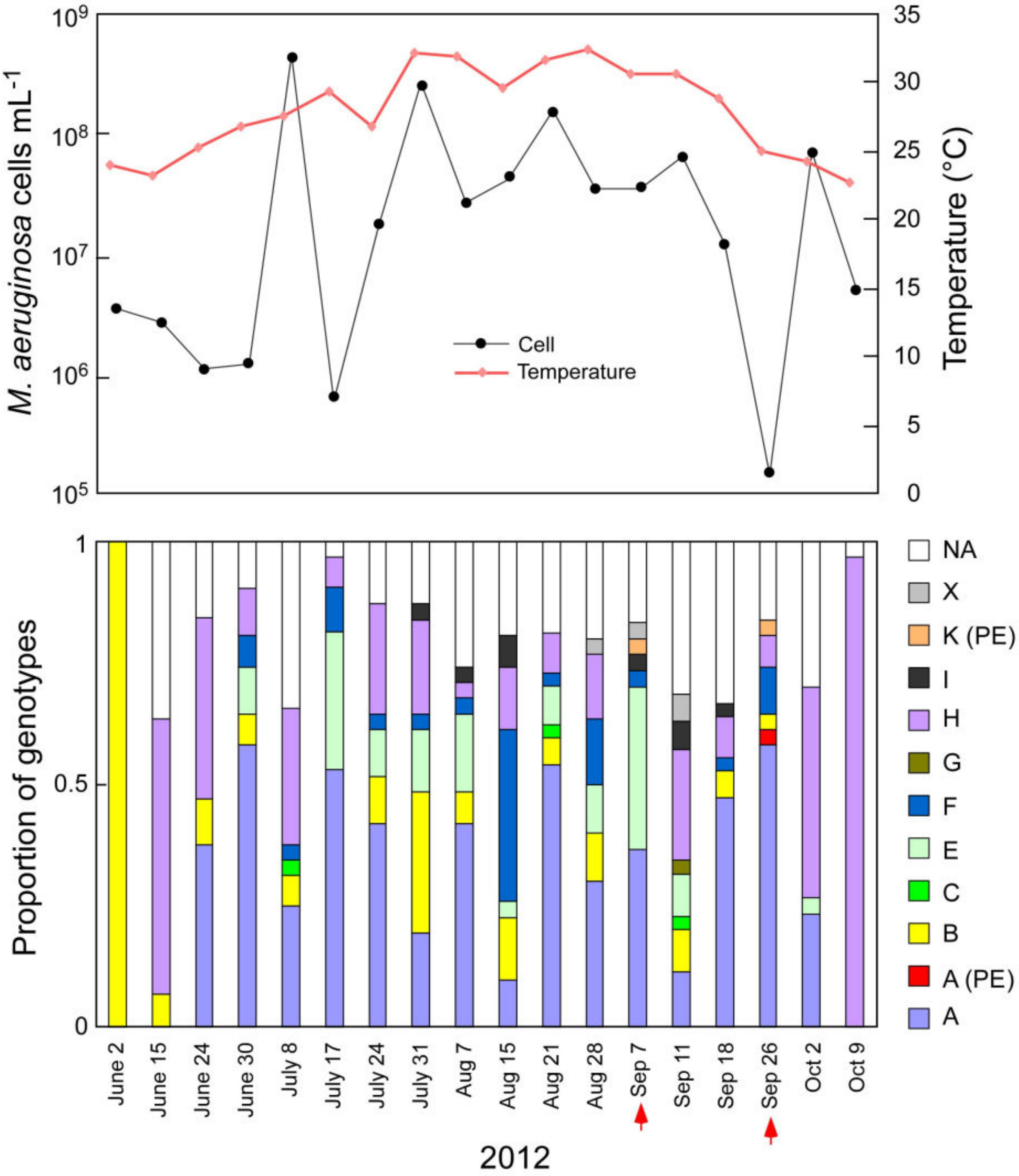

Fig. S3 Distribution of PE-containing genotypes in Lake Kasumigaura. Red arrows indicate the sampling times st which PE-containing genotypes were detected. The sampling location was identical to that from which NIES-2521 and NIES-4264 were isolated (see Materials and Methods). Genotype identification is based on clone library analyses (Tanabe *et al.*, 2018). At least 30 clones are included at every sampling time. PE-containing genotypes in Groups A and K can be identified by their specific *ftsZ* genotype (Data S1).
